# Exogenous interleukin 37 ameliorates atherosclerosis via inducing the Treg response in ApoE-deficient mice

**DOI:** 10.1038/s41598-017-02987-4

**Published:** 2017-06-12

**Authors:** Qingwei Ji, Kai Meng, Kunwu Yu, Song Huang, Ying Huang, Xiaohong Min, Yucheng Zhong, Bangwei Wu, Yuzhou Liu, Shaoping Nie, Jianwei Zhang, Yujie Zhou, Qiutang Zeng

**Affiliations:** 1 Department of Cardiology, Beijing Anzhen Hospital, Capital Medical University, Beijing Institute of Heart Lung and Blood Vessel Disease, The Key Laboratory of Remodeling-related Cardiovascular Disease, Ministry of Education, Beijing, 100029 China; 2 Laboratory of Cardiovascular Immunology, Institute of Cardiology, Union Hospital, Tongji Medical College, Huazhong University of Science and Technology, Wuhan, China; 3 Department of Orthopedics, Liyuan Hospital, Tongji Medical College, Huazhong University of Science and Technology, Wuhan, China; 4 Department of Ultrasound, the People’s Hospital of Guangxi Zhuang Autonomous Region, Nanning, 530021 China; 5 Department of Pathology, Puren Hospital, Wuhan University of Science and Technology, Wuhan, China; 6 Emergency & Critical Care Center, Beijing Anzhen Hospital, Capital Medical University, Beijing Institute of Heart Lung and Blood Vessel Disease, The Key Laboratory of Remodeling-related Cardiovascular Disease, Ministry of Education, Beijing, 100029 China

## Abstract

Our previous study indicated that interleukin (IL)-37 is involved in atherosclerosis. In the present study, Anterior tibial arteries were collected from diabetes patients and controls. A histopathological analysis showed that IL-37 was over-expressed in human atherosclerotic plaques. Many types of cells including macrophages, vascular smooth muscle cells (VSMCs), endothelial cells and T lymphocyte expressed IL-37 in human atherosclerotic plaques. ApoE**−/−** mice were divided into a control group and a recombinant human IL-37-treated group. The IL-37 treatment resulted in a significant decrease in macrophages and CD4+ T lymphocytes and a substantial increase in VSMCs and collagen in atherosclerotic plaques, resulting in a reduction in atherosclerotic plaque size. Furthermore, the IL-37 treatment modulated the CD4+ T lymphocyte activity, including a decrease in T helper cell type 1 (Th1) and Th17 cells and an increase in regulatory T (Treg) cells, and inhibited the maturity of dendritic cells both *in vivo* and *in vitro*. In addition, treatment with anti-IL-10 receptor monoclonal antibody abrogated the anti-atherosclerotic effects of IL-37. These data suggest that exogenous IL-37 ameliorates atherosclerosis via inducing the Treg response. IL-37 may be a novel therapeutic to prevent and treat atherosclerotic disease.

## Introduction

Atherosclerosis is a chronic inflammatory disease in the arterial wall that is initiated mainly in response to oxidized lipoproteins and controlled by immune system^1–3^. Both innate immunity, which is antigen- and memory-independent, and adaptive immunity, which is antigen- and memory-dependent, are involved in atherosclerosis^2^. The main cell type involved in innate immunity is macrophage. In contrast, T cells and dendritic cells (DCs) are the main cell types in adaptive immunity. Regulating the immune response has been considered as the therapeutic target for the treatment of atherosclerosis^4, 5^.

The novel anti-inflammatory cytokine interleukin (IL)-37 is a new member of the IL-1 family, and is only interleukin of this family that is absent in mice^6, 7^. The IL-1 family consists of 11 members, including IL-1α, IL-1β, IL-1Rα, IL-18, IL-33, IL-36Rα, IL-36α, IL-36β, IL-36γ, IL-37 and IL-38^8^. Most of the cytokines of the IL-1 family, such as IL-1 and IL-18, are pro-inflammatory cytokines and significantly promote the development of atherosclerosis^9^. Some, such as IL-1Rα and IL-33, are anti-inflammatory cytokines and efficiently ameliorate atherosclerosis in mice whereas the function of IL-36α remains unclear^9^. Evidence shows that both exogenous and endogenous IL-37 play a protective role in Inflammatory and autoimmune diseases in animal models via the inhibition of the generation of pro-inflammatory cytokines and the activation of macrophages and DCs^7, 10–14^. In addition, endogenous IL-37 has been found to induce the generation of tolerogenic DCs and regulatory T cells (Tregs) in skin contact hypersensitivity model^15^. These results demonstrated that IL-37 inhibits inflammation through modulating innate and adaptive immunity.

Recently, a clinical study from our group demonstrated that the plasma levels of IL-37 and the IL-37 expression in peripheral blood mononuclear cells were significantly increased in patients with acute coronary syndrome, which is the critical phase of coronary artery disease, compared to the control group^16^. Using a diabetic model, we found that recombinant human IL-37 attenuated both atherosclerosis and vascular calcification via regulating the production of osteoprotegerin and inflammatory cytokines such as IL-10, IL-18, TNF-α and IFN-γ^17^. These results firstly demonstrated the protective role of IL-37 in atherosclerosis. However, the mechanisms concerning the antiatherogenic effect of IL-37 still remain uncertain. Hence, we hypothesize that IL-37 may protect mice from atherosclerosis via modulating innate and adaptive immunity^17^. Firstly, human atherosclerotic plaques were collected to investigate whether IL-37 is expressed in plaques and which cell type is the source of IL-37. Then, ApoE**−/−** mice were treated with a recombinant human IL-37 protein to investigate the association of IL-37 with innate and adaptive immunity in the development of atherosclerosis.

## Results

### Over-expression of IL-37 in human atherosclerotic plaques

**Figure 1:**
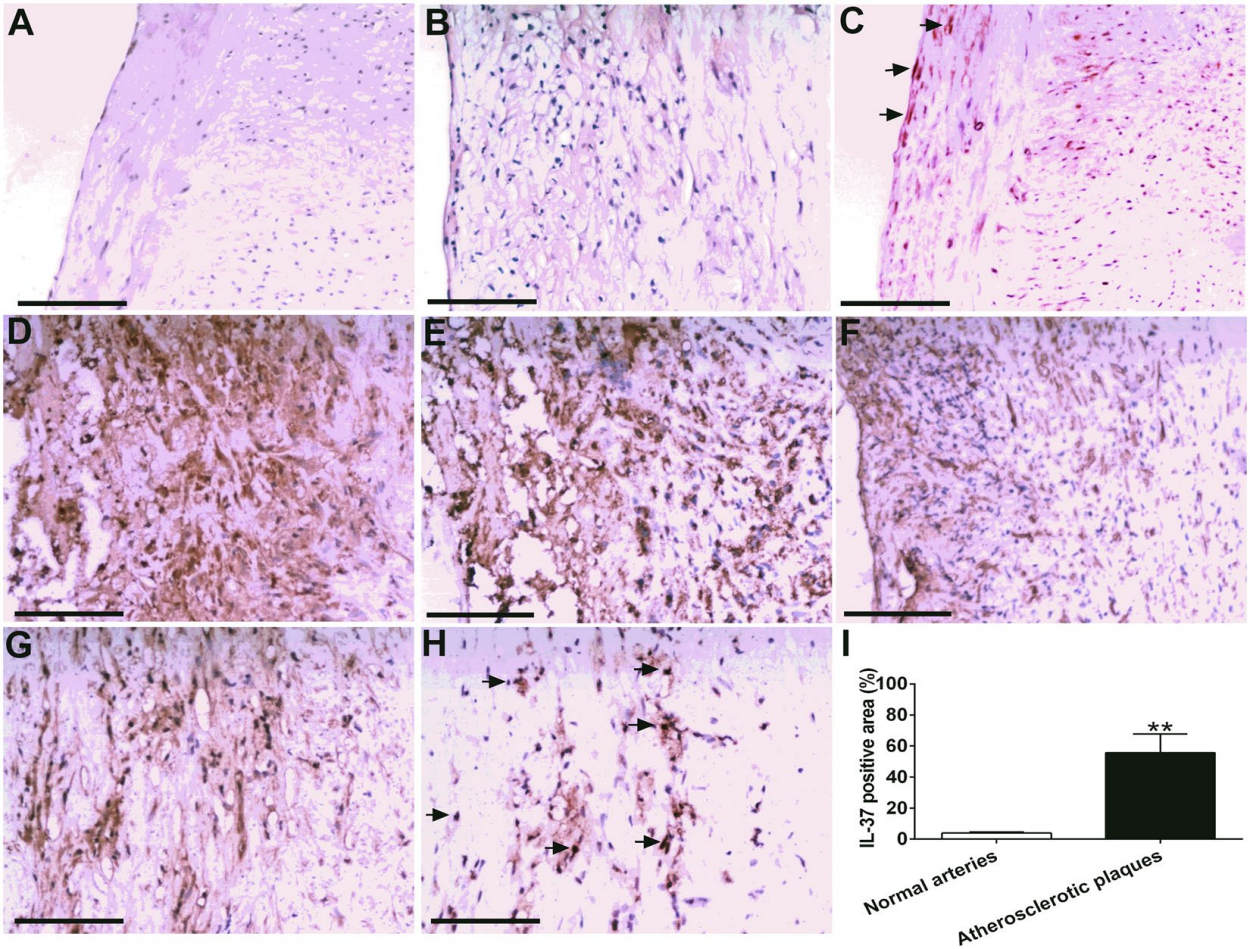
Over-expression of IL-37 in human atherosclerotic plaques. (**A**) Normal human anterior tibial artery stained with hematoxylin-eosin. (**B**) Human atherosclerotic plaque. (**C**) Little IL-37 expression in ECs and VSMCs in normal artery (indicated by arrowheads). (**D**) Abundant IL-37 expression in foam cells in atherosclerotic plaque. D through F, IL-37 expression (**D**) in macrophages identified by anti-CD68 antibody (**E**) and VSMCs identified by anti-α-SMA antibody (**F**). IL-37 expression (**G**) in T lymphocytes identified by anti-CD3 antibody (**H**). (**I**) Results of statistical analysis of IL-37 expression in normal arteries and atherosclerotic plaques. Values are presented as the means ± SEM, n = 8. Black bar = 200μm. **p < 0.01.

As shown in Fig. 1C, only a small quantity of IL-37 was detected in normal arteries in which it was mainly expressed in endothelial cells (ECs) and VSMCs. In contrast, abundant IL-37 was detected in atherosclerotic plaques in which it was mainly expressed in foam cells (Fig. 1D), which is consistent with a previous report^7^. We used anti-CD68 antibody and anti-α-SMA to identify macrophages and VSMCs in the plaque. The results showed that those IL-37-rich foam cells were macrophages and VSMCs (Fig. 1E and F, see Supplementary Fig. S1A and B, Fig. S2). Anti-CD3 antibody was used to identify T lymphocytes and the results showed that IL-37 was also expressed by T lymphocytes (Fig. 1G and H, see Supplementary Fig. S1C). In addition, IL-37-positive area was significantly higher in atherosclerotic plaques than normal arteries (55.6 ± 24.5% vs 3.9 ± 1.2%, P < 0.01) (Fig. 1I).

### IL-37 ameliorates atherosclerosis in ApoE−/−mice

The results of body weight and plasma lipid analysis, including the total plasma cholesterol, high-density lipoprotein cholesterol, and triglycerides, were similar in the two groups (Table 1). These results indicated that the administration of IL-37 had no obvious effect on body weight or plasma lipid levels.

**Table 1:** Body weight and lipid levels in all groups.

	Age	Control	IL-37
Body weight (g)	8 weeks	20.7 ± 0.3	20.9 ± 0.2
Body weight (g)	18 weeks	29.7 ± 0.4	29.9 ± 0.3
Total cholesterol (mg/dl)	18 weeks	658.9 ± 23.2	672.4 ± 28.7
HDL cholesterol (mg/dl)	18 weeks	56.5 ± 5.4	52.4 ± 4.9
Triglycerides (mg/dl)	18 weeks	853.6 ± 46.2	844.2 ± 43.1

Note: Values are presented as the mean ± SEM, n = 12.

**Figure 2:**
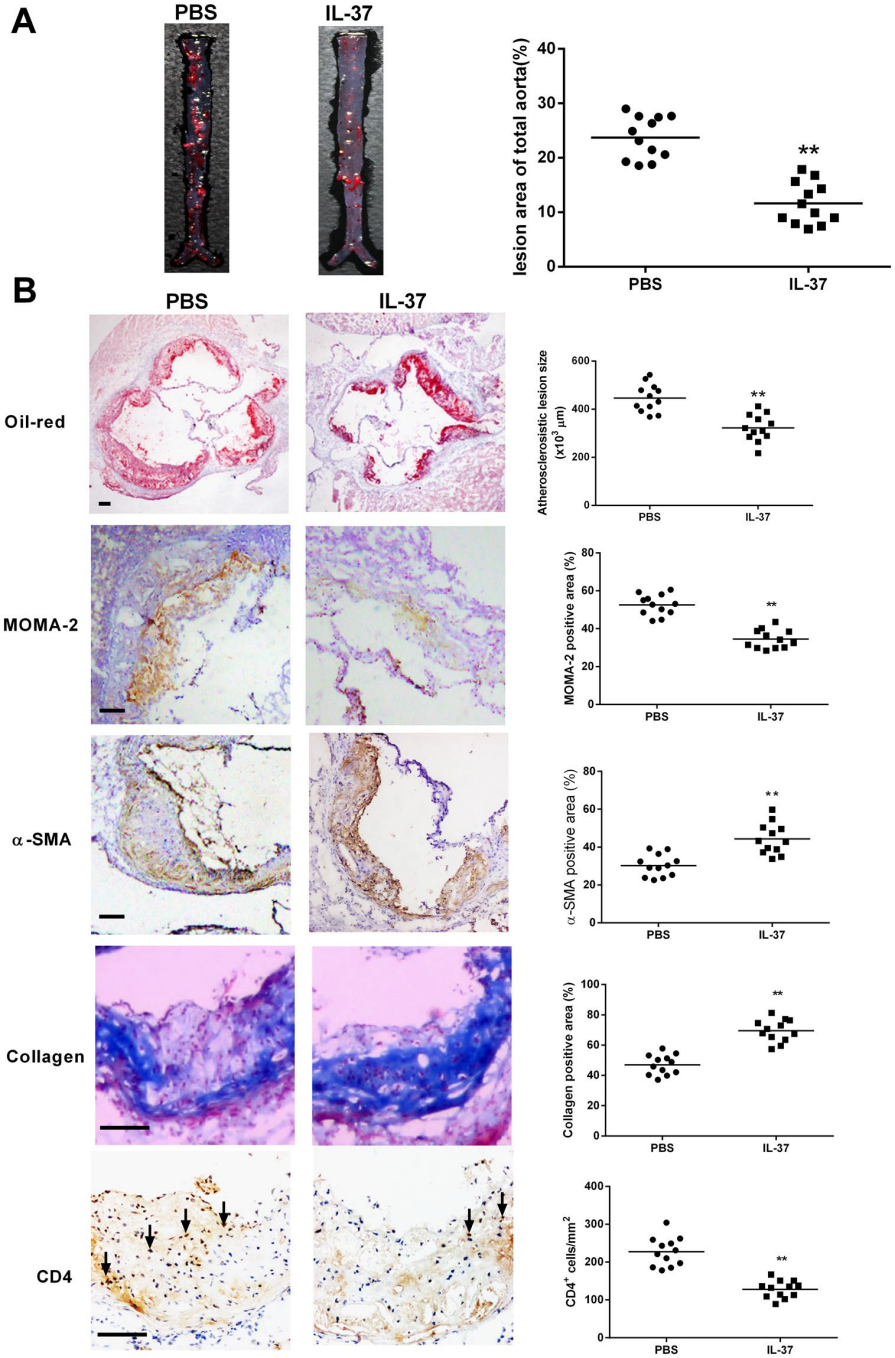
IL-37 ameliorates the development of atherosclerosis. (**A**) Representative photographs of Oil Red O staining in the surface lesion area of the entire aorta, and quantitative analysis of the percent lesion area of the entire vessel area (*p < 0.05). (**B**) Representative sections of aortic sinus stained with Oil Red O (Black bar = 200 μm), antibodies to MOMA-2 (Black bar = 200 μm), α-SMA (Black bar = 200 μm), collagen (Black bar = 200 μm) and CD4 (Black bar = 200 μm) staining in the two groups (Black bar = 200 μm), and quantitative analysis of the lesion size of aortic sinus, MOMA-2, α-SMA, collagen and CD4 staining in the two groups. MOMA indicates monocytes/macrophages; SMA, smooth muscle actin. Values are presented as the means ± SEM, n = 12. **p < 0.01.

To investigate the role of IL-37 in protecting against atherosclerosis, we detected the atherosclerosis lesion sizes between the IL-37 group and the control group. The results showed that the atherosclerosis lesion size in the aortic sinus of the IL-37 group was significantly decreased compared to that of the control group (3.22 ± 0.16 × 10^5^ μm^2^ versus 4.46 ± 0.17 × 10^5^ μm^2^, P < 0.01; Fig. 2B). In addition, we also quantified the lesion area in the en face preparations of the whole aorta. Our analysis revealed that there was a significant reduction in the aortic plaque burden in the IL-37 group compared to the control group (11.63 ± 1.11% versus 23.72 ± 1.12%, P < 0.01; Fig. 2A). Furthermore, we performed MOMA-2, α-SMA, Masson as well as CD4 staining of the plaques in the two groups. The macrophages staining in the IL-37 group was decreased compared to the control group (34.49 ± 1.42% versus 52.59 ± 1.58%, P < 0.01; Fig. 2B). The α-SMA positive area in the IL-37 group was increased compared to the control group (44.33 ± 2.35% versus 30.26 ± 1.69%, P < 0.01; Fig. 2B). Masson staining for collagen in the IL-37 group was increased compared to the control group (69.52 ± 2.11% versus 47.08 ± 1.90%, P < 0.01; Fig. 2B). The CD4 staining in the IL-37 group was decreased compared to the control group (128.10 ± 6.62 cell/mm2 versus 227.10 ± 11.01 cells/mm^2^ for CD4+ T cells, P < 0.01; Fig. 2B).

### IL-37 inhibits the maturation of DCs ***in vivo*** and ***in vitro***

**Figure 3:**
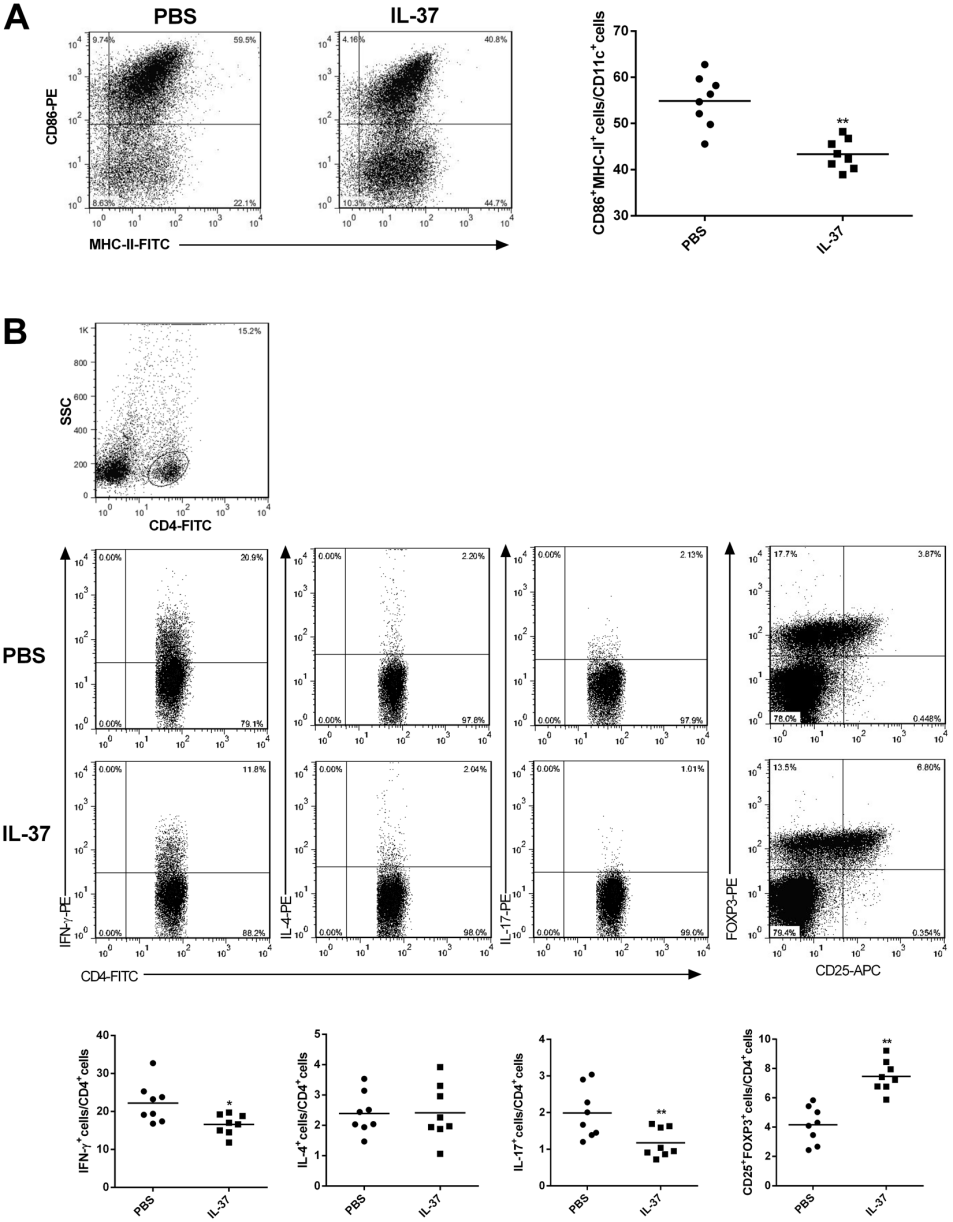
Effect of IL-37 administration on DCs and CD4^+^T cells in spleen of ApoE**−/−** mice. (**A**) Flow cytometry results of DCs (CD86+MHC-II+CD11c+) are shown. The numbers in upper right quadrants indicate positive percentages of these cells. Results of statistical analysis of DCs. (**B**) Flow cytometry results of Th1 (CD4+IFN-γ+), Th2 (CD4+IL-4+), Th17 (CD4+IL-17+), and Tregs (CD4+CD25+FOXP3+) are shown. The numbers in upper right quadrants indicate positive percentages of these cells. Results of statistical analysis of Th1 cells, Th2 cells, Th17 cells, and Tregs. Values are presented as the means ± SEM, n = 8. *p < 0.05, and **p < 0.01.

To assess the phenotype of splenic DCs, we analyzed splenic single-cell suspensions by flow cytometry. The data revealed that CD11c + DCs express decreased levels of CD86 and MHC-II in the IL-37 group compared to the control group (43.34 ± 1.16% versus 54.89 ± 1.98%, P < 0.01, Fig. 3A).

**Figure 4:**
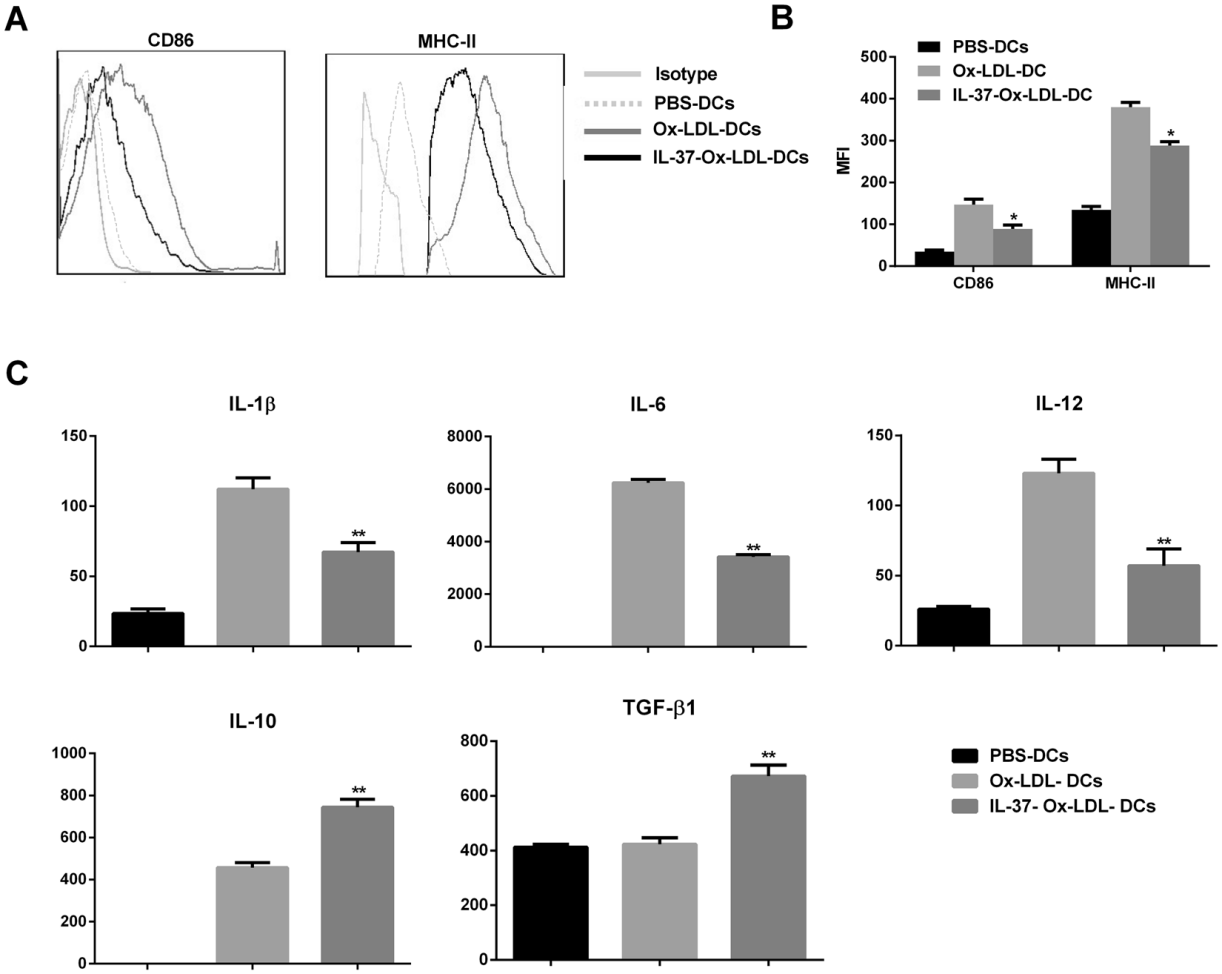
Effect of IL-37 on the maturation of BMDCs *in vitro*. (**A**) Flow cytometry results of CD86 and MHC-II. (**B**) Representative MFI of CD86 and MHC-II in the cultured BMDCs. (**C**) Results of statistical analysis of the cytokine levels in the cultured supernatant. The data represent 3 experiments in each group. Values are presented as the means ± SEM. *p < 0.05, and **p < 0.01.

OxLDL plays an important role in atherosclerosis and can promote dendritic cells to mature. We used oxLDL and LPS as a positive control, and found 20 µg/ml of ox-LDL was not toxic for DCs (see Supplementary Figs S2 and S3). To investigate the effect of IL-37 on the maturation of DC *in vitro*, we analyzed their phenotypes. The DCs treated with IL-37 plus oxLDL displayed distinct downregulation in the mean fluorescence intensity of the costimulatory factor CD86 (89.2 ± 8.9 versus 147.2 ± 13.1, P < 0.01; Fig. 4A and B) and MHC-II (379.7 ± 11.8 versus 288.5 ± 9.2, P < 0.01; Fig. 4A and B) compared to those DCs treated with oxLDL. We also detected the cytokine secretion of DCs. As shown in Fig. 4C, the secretions of IL-1β, IL-6 and IL-12 were significantly lower in the DCs treated with oxLDL plus IL-37 (40%, 45%, and 54% reduction, respectively) compared to those treated with oxLDL. In addition, the secretions of IL-10 and TGF-β1 were enhanced in the DCs treated with oxLDL plus IL-37 (62% and 59% increment, respectively) compared to those treated with Ox-LDL.

### IL-37 modulates the polarization of CD4+ T lymphocyte ***in vivo*** and ***in vitro***

To investigate whether IL-37 modulates the polarization of CD4+ T lymphocyte *in vivo*, we detected the different lymphocytes of the spleen in the two groups. As shown in Fig. 3B, the frequencies of the Th1 cells (CD4 + IFN-γ + T cells) and the Th17 cells (CD4 + IL-17 + T cells) were significantly decreased in the IL-37 group compared to the control group (16.58 ± 0.96% versus 22.21 ± 1.86% for Th1, P < 0.01; 1.17 ± 0.14% versus 1.99 ± 0.35% for Th17, P < 0.01). In contrast, the frequencies of Tregs (CD4 + CD25 + FOXP3 + T cells) were significantly increased in the IL-37 group compared to the control group (7.41 ± 0.59% versus 4.10 ± 0.57%, P < 0.01). Interestingly, there was no significant difference in Th2 cells between the two groups.

**Figure 5:**
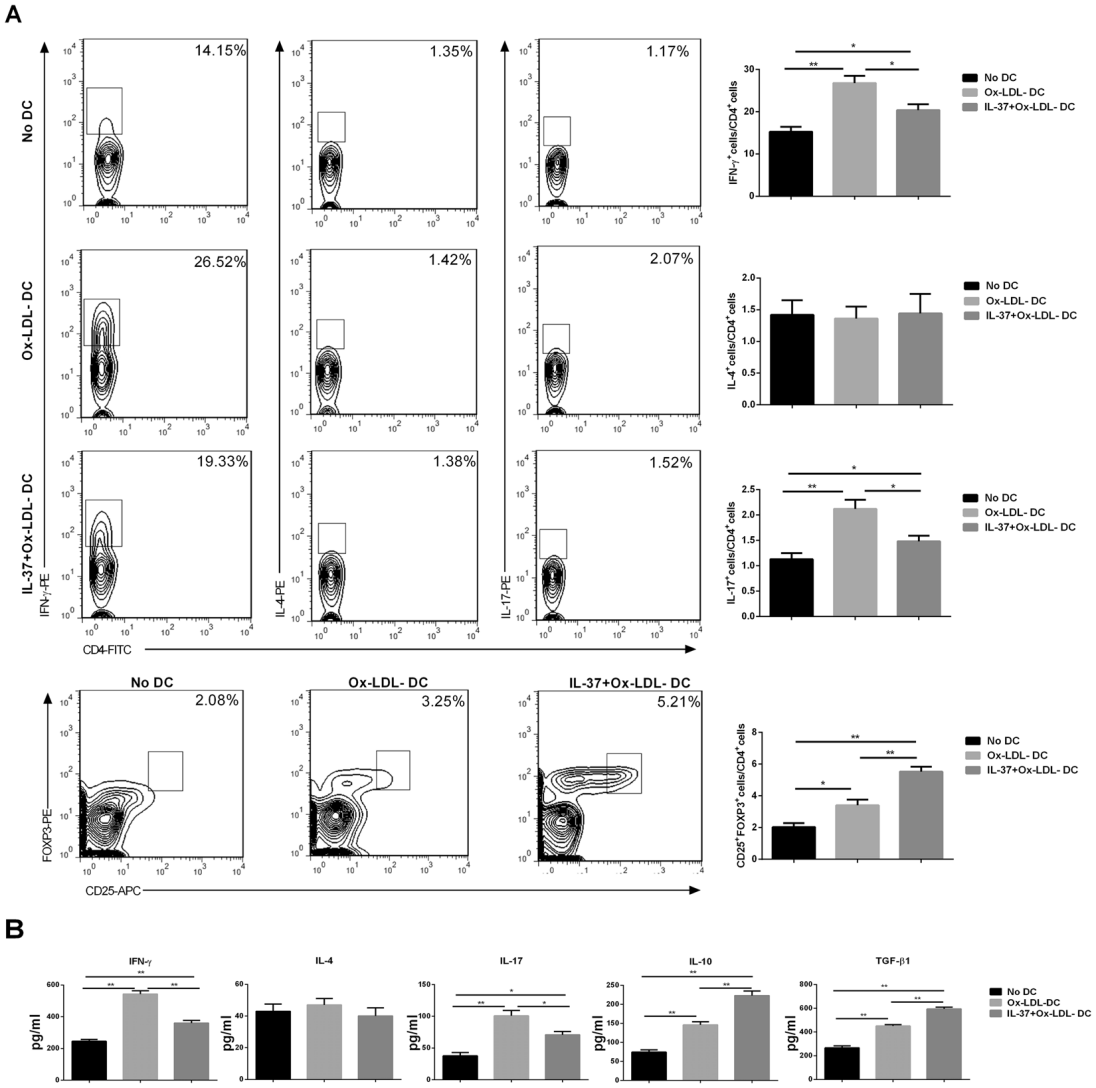
Effect of IL-37 on the Th1/Th2/Th17/Treg paradigm *in vitro*. (**A**) Flow cytometry results of (CD4+IFN-γ+), Th2 (CD4+IL-4+), Th17 (CD4+IL-17+), and Tregs (CD4+CD25+FOXP3+) are shown. Results of statistical analysis of Th1 cells, Th2 cells, Th17 cells, and Tregs. (**B**) Results of statistical analysis of the cytokine levels in the cultured supernatant. The data represent 3 experiments in each group. Values are presented as the means ± SEM. *p < 0.05, and **p < 0.01.

To observe the regulatory effects of IL-37 on the Th1/Th2/Th17/Treg paradigm *in vitro*. We cultured CD4+ T cells separately with BMDCs that were treated with oxLDL or oxLDL plus IL-37. As shown in Fig. 5A, the CD4+ T cells that were activated in the presence of oxLDL plus IL-37-DCs contained a reduced number of Th1 and Th17 cells than those that were activated with oxLDL-DCs (20.35 ± 1.41% versus 26.78 ± 1.69% of Th1, P < 0.05; 1.48 ± 0.11% versus 2.12 ± 0.18% of Th17, P < 0.05); they also contained an increased number of Treg cells (5.53 ± 0.31%) than those that were activated with oxLDL-DCs (3.41 ± 0.36%, P < 0.01) and without DCs (2.03 ± 0.25%, P < 0.01). In addition, the cytokines of the CD4+ T cells that were co-cultured with the oxLDL plus IL-37-DCs had lower amounts of IFN-γ and IL-17 but greater amounts of TGF-β1 and IL-10 (Fig. 5B). Additionally, there were no differences in the number of Th2 cells and the amount of IL-4 in the three groups (Fig. 5A and B).

### IL-37 attenuates both systemic and local inflammation

**Figure 6:**
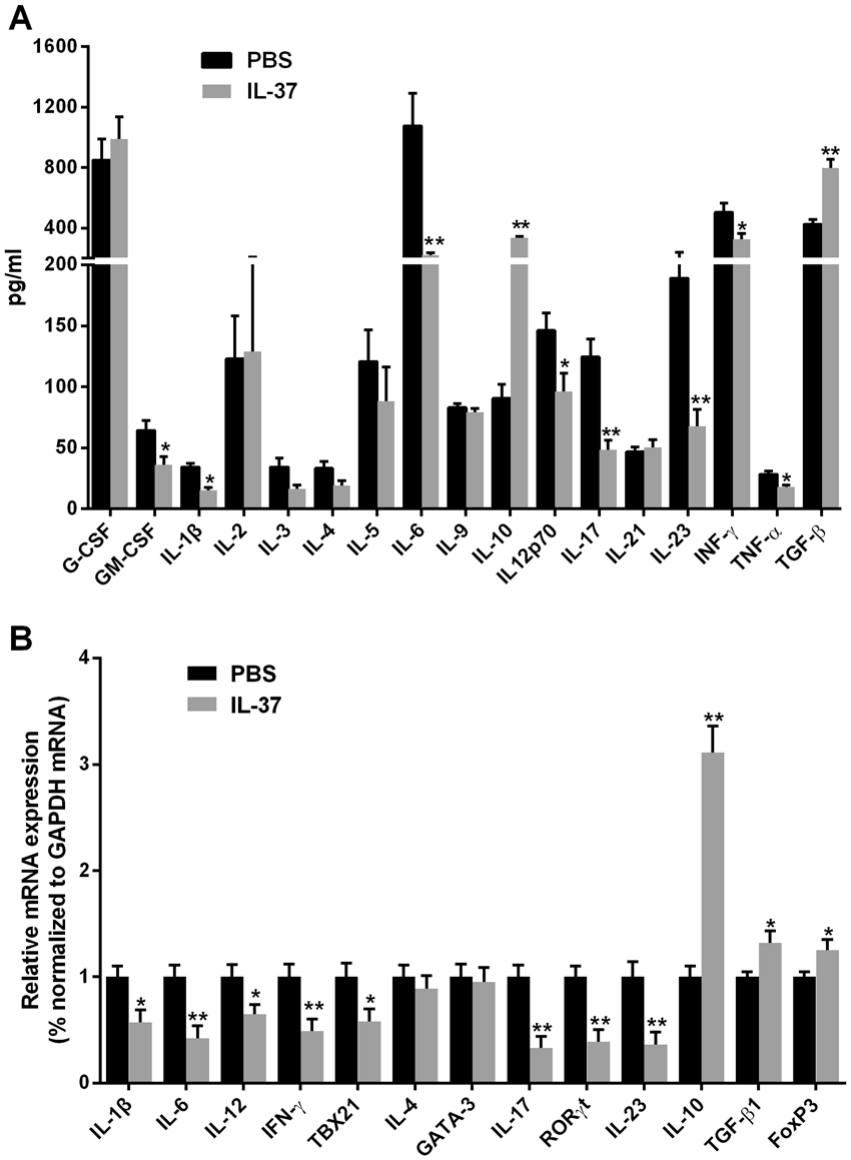
The levels of plasma cytokines in the two groups and the mRNA expression in the aorta. (**A**) The levels of plasma G-CSF, GM-CSF, IL-1β, IL-2, IL-3, IL-4, IL-5, IL-6, IL-9, IL-10, IL-12p70, IL-17, IL-21,IL-23, IFN-γ, TNF-α and TGF-β1 in the two groups. (**B**) Expression of IL-1β, IL-4, IL-6, IL-10, IL-12p70, IFN-γ, TBX21, RORγT, IL-17, IL-23, GATA-3, TGF-β1 and FOXP3 mRNA in aorta in the two groups. Values are presented as the means ± SEM, n = 5 to 7. *p < 0.05, and **p < 0.01.

To examine the effect of the administration of IL-37 on systemic inflammation, we analyzed the cytokine levels in the plasma between the IL-37 group and the control group. As shown in Fig. 6A, the IL-37-treated mice showed lower levels of GM-CSF, IL-1β, IL-6, IL-12p70, IL-17, IL-23, IFN-γ and TNF-α but higher levels of IL-10 and TGF-β1 compared to the control group. There was no significant difference in G-CSF, IL-2, IL-3, IL-4, IL-5, IL-9, or IL-21 levels between the two groups. In addition, we analyzed the mRNA expression of these cytokines in the aorta of the mice. Our results showed that the IL-37-treated mice showed lower levels of IL-1β, IL-6, IL-12, IL-17, IFN-γ, TBX21, and RORγt but higher levels of IL-10, TGF-β1 and FOXP3 than the control group (Fig. 6B).

### Anti-IL-10 treatment abrogates beneficial effects of IL-37 on atherosclerosis

**Figure 7:**
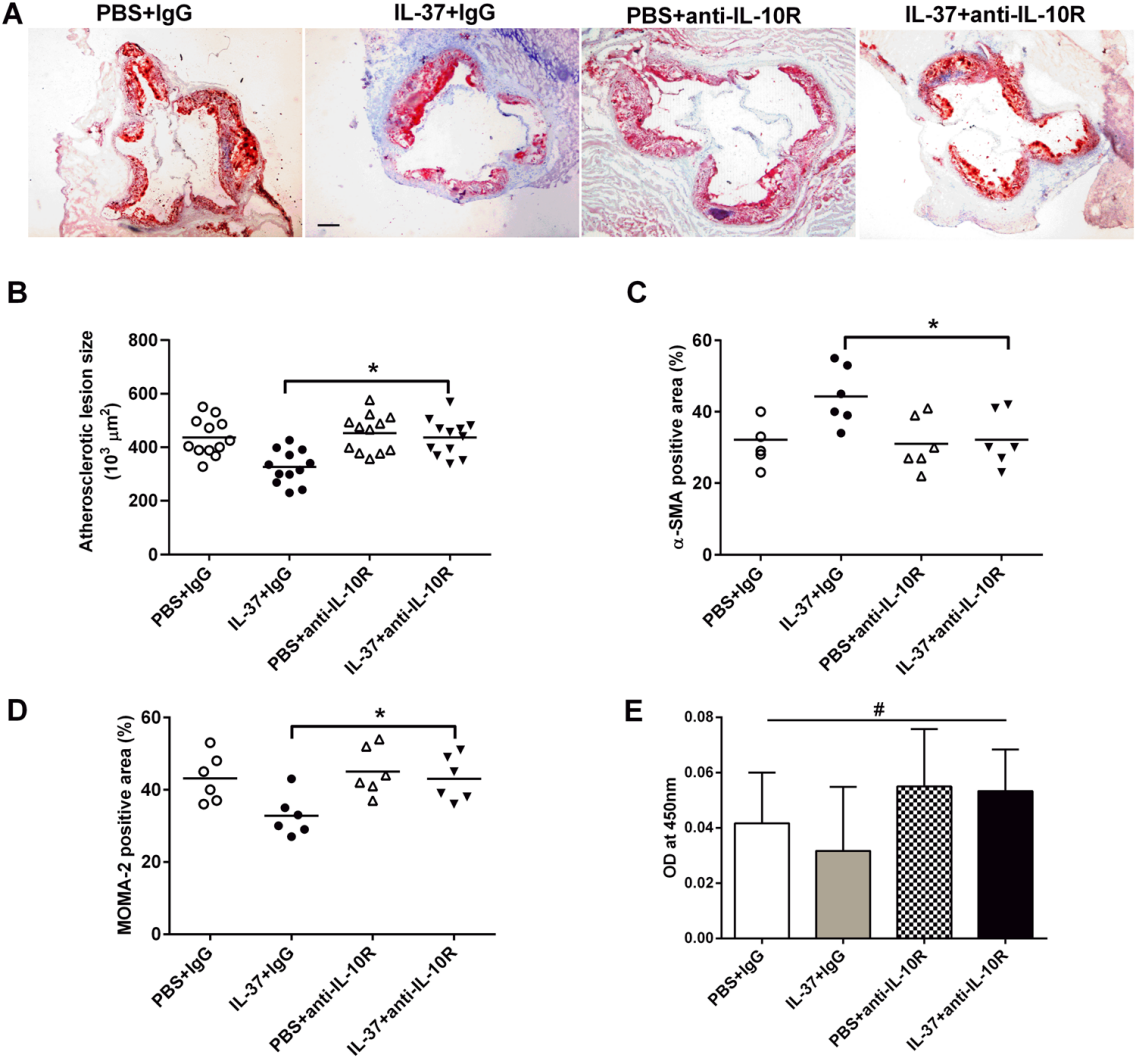
Anti-IL-10 treatment abrogates beneficial effects of IL-37 on atherosclerosis. (**A**) Representative sections of aortic sinus stained with Oil Red O staining in ApoE**−/−** mice at 18 weeks. Black bar = 200 μm. n = 12. (**B**) Through (**D**) Quantitative analysis of lesion size, MOMA-2, and α-SMA staining. (**E**) Levels of plasma neutralizing antibodies of anti-IL-10R IgG. Values are presented as the mean ± SEM, n = 6. MOMA indicates monocyte/macrophage; SMA, smooth muscle actin. *p < 0.05, and **p < 0.01.

In order to assess the role of IL-10 in IL-37–mediated protection from atherosclerosis, we administered IL-37 or PBS together with anti-IL-10R mAb or isotype antibody. The mice treated with anti-IL-10R mAb and IL-37 developed a significantly lager atherosclerotic lesion size in the aortic sinus compared to the IgG and IL-37-treated mice (4.37 ± 0.20 × 10^5^ μm^2^ versus 3.26 ± 0.18 × 10^5^ μm^2^, P < 0.01; Fig. 7A and B). We also found significant difference in staining of atherosclerotic lesion macrophages and α-SMA between the two groups (P < 0.05, Fig. 7C and D). There were no significant differences in atherosclerotic lesion size between anti-IL-10R mAb and PBS group and IgG and PBS group (P > 0.05, Fig. 7A and B). Levels of plasma neutralizing antibodies of anti-IL-10R IgG or IL-37 were almost undetectable in all groups (Fig. 7E, Fig. S5).

## Discussion

IL-37, found in 2000, is the only IL-1 family member absent in mice^7, 18, 19^. IL-37 has five splice variants (IL-37a–e), and IL-37b (also known as IL-37) is the best characterized IL-37 isoform and the main IL-37 isoform present in peripheral blood^7^. The anti-inflammatory properties of IL-37 were unknown until Nold *et al*. discovered in 2010 that endogenous IL-37 was effectively induced by inflammatory stimulation, thereby inhibiting the production of pro-inflammatory cytokines such as IL-6, TNF-α and IL-1β, and protecting mice from lipopolysaccharide (LPS)-induced shock^7^. Because endogenous IL-37 has powerful anti-inflammatory properties and significantly ameliorates intestinal inflammation in a mouse colitis model, the researchers hypothesized that the mechanism of action of IL-37 was likely intracellular; therefore, the exogenous administration of IL-37 could not perform the anti-inflammatory functions^20^. In addition, the endogenous IL-37 has potent immunoregulatory properties that suppress skin inflammation by inducing the generation of tolerogenic DCs and Tregs^15^. However, accumulating evidence has shown that not only endogenous IL-37 but also exogenous IL-37 has anti-inflammatory properties via binding to its receptor SIGIRR and IL-18Rα^10, 14^. We and other researchers reported that treatment with recombinant human IL-37 reduced the production of pro-inflammatory cytokines, chemokines and reactive oxygen species (ROS), and withdrew neutrophil infiltration, resulting in the amelioration of I/R injury^11, 12^. In addition, we firstly demonstrated that exogenous IL-37 protected mice from atherosclerosis in a diabetic model^21^. However, whether IL-37 regulates immune response in atherosclerosis remains uncertain.

The article and figure about IL-37 in human atherosclerotic plaques has not been published, although a review firstly revealed that IL-37 was detected in the foam-like cells of atherosclerotic coronary and carotid artery plaques^7^. Therefore, we detected the expression of IL-37 in human anterior tibial arterial specimens. Using immunocytochemical staining methods, we found that IL-37 was highly expressed in human atherosclerotic plaques but was rarely expressed in the normal arterial wall. Importantly, the results showed that multiple cell types including ECs, foam-like cells, macrophages and VSMCs expressed the IL-37 protein, and macrophages and VSMCs were the main source of IL-37. In previous studies, IL-37 was not found in blood lymphocytes in human by using anti-IL-37b polyclonal antibody, although it expressed in thymus, spleen and lymph nodes^6, 7, 22^. However, the results from our study demonstrated that T lymphocytes expressed the IL-37 protein in human atherosclerotic plaques. The results were consistent with Teng s report^23^, in which IL-37 was found to be expressed by T cells and macrophages in human psoriatic plaques. Hence, our present data firstly indicated that IL-37 is involved in innate and adaptive immunity in human atherosclerosis.

To investigate the effect of IL-37 on atherosclerosis, we treated ApoE^−/−^ mice with recombinant human IL-37 protein. The results demonstrated that IL-37 treatment led to a significant reduction in the size of the atherosclerotic plaque accompanied with a decrease in macrophage infiltration. Accumulating evidence has demonstrated that activated macrophages accumulate in atherosclerotic lesions and play an indispensable role throughout the different stages of atherosclerosis, from the occurrence of fatty streaks to plaque rupture and thrombosis^24^. Macrophages not only express scavenger receptors, resulting in the uncontrolled uptake of oxLDL and the formation of foam cells, but also act as antigen presentation cells to promote T lymphocyte activation, exacerbating the process of atherosclerosis; therefore, macrophage is vital for innate and adaptive immunity. The inhibition of macrophage activation is regarded as a therapeutic approach in atherosclerotic disease. Evidence has demonstrated that regulating the activation of macrophages is the main mechanism by which IL-37 controls inflammation and attenuates autoimmune and inflammatory diseases including I/R injury^11, 12^, obesity^13^ and colitis^20^. Consistent with these studies, the present study indicated that IL-37 treatment attenuated atherosclerosis by inhibited the infiltration of macrophages, suggesting a protective mechanism of IL-37 in atherosclerosis.

The polarization of DCs and CD4^+^ T cells is critical for the development of atherosclerosis. Accumulating evidence has demonstrated that mature DCs, Th1 and Th17 cells possess potential pathogenic properties whereas tolerogenic DCs and Treg effectively ameliorate atherosclerosis^2–5, 25–29^. Previous studies have shown that IL-37 significantly inhibited the LPS-induced DC activation and led to a reduction in the expression of CD86 and MHC II on DCs^7^, which was also elicited in our animal experiment. In addition, cultured DCs exhibited a decrease in CD86 and MHC II expression and an increase in IL-10 and TGF-β1 production after IL-37 treatment, suggesting that exogenous IL-37 may induce the generation of tolerogenic DCs. Furthermore, exogenous IL-37 not only promoted the Treg response but also suppressed the Th1 and Th17 responses *in vivo* and *in vitro*, and these effects were related to the inhibition of DC maturation. Consistent with these effects, the IL-37 treatment significantly increased the contents of VSMCs and collagen in atherosclerotic plaques, suggesting that IL-37 promotes plaque stability beyond lesion size control. Together, these findings indicate that the protection imparted by IL-37 is mediated by polarization of the adaptive immune system.

The results from ELISA and RT-PCR examinations also supported the immunoregulatory properties of IL-37 in atherosclerosis. We measured the serum level of eighteen cytokines in the present study. The levels of Th1-relatived cytokines (IL-12p70, IFN-γ and TNF-α) and Th17-relatived cytokines (IL-6, IL-17 and IL-23) were significantly decreased after IL-37 treatment. However, IL-37 treatment did not influence the production of Th2-relatived cytokines (IL-4 and IL-5). In the last two decades, numerous studies have established the atherogenic role of most of these cytokines such as IL-1β, IL-6, IL-12p70, IL-17, IFN-γ and TNF-α^2, 3, 10, 28, 29^. Although no evidence has confirmed the direct atherogenic role of IL-23, IL-23 has been found to be critical for the pro-atherosclerotic effects of GM-CSF^30^, which was increased significantly in our present study. In addition, both IL-1β and GM-CSF have been found to promote Th1 and Th17 immune response^31–34^. Our present study showed that IL-37 treatment significantly suppressed the production of IL-1β and GM-CSF, suggesting an indirect mechanism of IL-37 modulating the Th1 and Th17 immune response. In addition, the RT-PCR examination not only showed the consistent results in these cytokines but also exhibited a reduction in the expression of Th1 and Th17-relatived transcription factors (TBX21 and RORγt, respectively) after IL-37 treatment. Therefore, the ELISA and RT-PCR analysis also indicated that IL-37 may ameliorate atherosclerosis through dampening the Th1 and Th17 immune response.

In addition to effects on pro-inflammatory cytokine production, we found that exogenous IL-37 promoted a significant increase in the production of anti-inflammatory cytokines IL-10 and TGF-β1, which was also shown in our previous study^12^. Although many cell types secret IL-10 and TGF-β1, Treg secrets abundant of IL-10 and TGF-β1and the two cytokines are critical for the generation and function of Treg; therefore, IL-10 and TGF-β1 have been considered as the effector cytokines of Treg. Both IL-10 and TGF-β1 have been confirmed to exhibit antiatherogenic properties using different approaches including transfer gene models, knockout models, neutralization, and adenovirus expression vector^2, 3, 9^. In our previous studies, we also found that TGF-β plays a vital role in an oxLDL and thymic stromal lymphopoietin (TSLP) treated atherosclerosis prone model^25, 26, 35^. Because not blocking TGF-β1 signaling but blocking IL-10 signaling significantly abolished the protective effects of IL-37 in a myocardial I/R injury model^12^, and because the ascending range of IL-10 was more significant than TGF-β1 in the present study, the anti-IL-10R mAb was used to clarify whether IL-10 is indispensable for the protective role of IL-37 in atherosclerosis. The results showed that the atherosclerosis lesion size in the aortic sinus of the IL-37 plus anti-IL-10R mAb group was significantly increased compared to that of the IL-37 plus isotype antibody group, however, anti-IL-10R mAb treatment did not increase the atherosclerosis lesion size in PBS group, suggesting that IL-10 is indispensable for the antiatherogenic effects of IL-37, which is consistent with our previous study^10^. Indeed, previous studies demonstrated that IL-10 transgene or IL-10 knockout could significantly inhibit or increase the development of atherosclerosis^2, 3, 9^, we infer that a slight effect of IL-10R neutralizing antibodies on atherosclerosis in PBS group was caused by: 1) IL-10 knockout but not IL-10R antibodies could inhibit IL-10 pathway completely, 2) a significant increased IL-10 by both atherosclerotic inflammation and IL-37 but not atherosclerotic inflammation alone respond to IL-10R antibodies well. However, McNamee *et al*. found that IL-10 was not required for the anti-inflammatory effects of IL-37 because anti-IL-10R antibody did not reverse IL-37-mediated protection, although IL-10 level was increased 6-fold in that study^20^. Hence, the different disease models may be contributed to this discrepancy in the three studies.

In conclusion, the data presented in this study demonstrate that abundant IL-37 is expressed in multiple cell types in human atherosclerotic plaques and that the administration of recombinant human IL-37 significantly reduces atherosclerotic plaque size, accompanied by a regulation in innate and adaptive immunity. In addition, the Treg response is indispensable for the antiatherogenic effects of IL-37. Therefore, the present study provides the direct evidence indicating the anti-atherogenic role of IL-37 and suggesting that IL-37 may be a novel therapeutic to prevent and treat atherosclerotic disease. There are some limitations in the present study. To imitate the change in the expression of endogenous IL-37 during the development of human atherosclerosis and elucidate the effects of endogenous IL-37 on atherosclerosis, future studies using IL-37 transgenic mice and other IL-37-related molecular knockout mice, such as SIGIRR knockout mice, should be performed.

## Methods

### Antibodies and reagents

The recombinant human IL-37 was purchased from Adipogen AG (Liestal, Switzerland). The anti-mice IL-10R mAb and control rat IgG_2A_ were purchased from R&D Systems (Minneapolis, MN, USA). The splenocytes and DCs were cultured in complete RPMI 1640 (Gibco) supplemented with 10% FCS (Gibco) and 100 U/ml streptomycin/penicillin. GM-CSF (lot no. 05075 5-1) and IL-4 (lot no. 1106CY4 9) were obtained from Peprotech (Rocky Hill, NJ). Anti-mice CD4-FITC antibody, anti-mice CD25-APC antibody, anti-mice interferon (IFN)-γ-PE antibody, anti-mice IL-4-PE antibody, anti-mice IL-17A-PE antibody, and anti-mice FOXP3-PE antibody were obtained from eBioscience (San Diego, CA). Purified anti-mice CD4 antibody (clone RM4-5) was from BD Systems. Anti-mice MOMA-2 antibody (lot no. NG1904267) was purchased from Millipore, and anti-mice α-SMA was obtained from Abcom. Nonspecific identical isotype control (rat IgG obtained from Abcom) antibodies were used as negative controls. The Quantibody Mouse Interleukin Array 1 was purchased from RayBiotech (Norcross, GA). IFN-γ, IL-1β, IL-4, IL-6, IL-10, IL-12p70, IL-17 and TGF-β1 ELISA kits were purchased from eBioscience. Anti-human IL-37 antibody (lot no. GR118844-1) was purchased from Abcom. Anti-human CD3 antibody (lot no. SP7), anti-human CD68 antibody (lot no. PGM1) and anti-human α-SMA antibody (lot no. 1A4) were purchased from Thermo Fisher Scientific Anatomical Pathology (Shanghai, China).

### Human tissue samples preparation and immunohistochemistry

In our previous study, we found that circulating IL-37 levels were elevated in patients with coronary artery disease^16^. We investigated whether IL-37 expression was elevated in human atherosclerotic plaques in the present study. Anterior tibial arterial wall specimens containing atherosclerotic plaques were obtained from 8 patients with type 2 diabetes mellitus (T2DM) (5 men, 3 women; age ± SD: 67 ± 11 years; range: 50 to 80 years) who were amputated in the Department of Orthopedics, Liyuan Hospital (clinical characteristics of patients showed in Table 2). Anterior tibial arterial specimens free of atherosclerosis were obtained from 8 patients suffering from a traffic accident. After surgery, the samples were fixed in formalin and embedded in paraffin for histology. For the tissues, the sections were routinely stained with hematoxylin-eosin or with anti-human IL-37 antibody. To determine the cellular localization of IL-37, anti-CD3 antibody, anti-CD68 antibody and anti-α-SMA antibody were used to identify T cell, macrophage and vascular smooth muscle cells (VSMCs) in atherosclerotic plaque. For the quantitative analysis of immunohistochemistry, images were visualized and analyzed using the HMIAS Series Color Medical Image Analyze System (Champion Image Ltd., China).

**Table 2:** Table Clinical characteristics of patients.

Characteristics	Control	Diabetes	P value
Age (years)	37 ± 12	67 ± 11	NS
Sex (male/female)	5/3	6/2	NS
Hypertension, n (%)	0	6(75)	<0.05
CHD, n (%)	0	1(12.5)	NS
Smoking, n (%)	2 (25)	2(25)	NS
BMI (Kg/m^2^)	25.7 ± 3.8	28.4 ± 3.1	NS
SBP (mmHg)	113 ± 15	133 ± 24	NS
DBP (mmHg)	73 ± 8	82 ± 17	<0.05
GLU (mmol/L)	4.93 ± 0.46	9.15 ± 2.70	<0.05
HbA1C (%)	4.82 ± 0.46	7.31 ± 1.24	<0.05
TG (mmol/L)	1.52 ± 0.79	2.00 ± 2.48	NS
TC (mmol/L)	3.90 ± 0.95	3.95 ± 0.94	NS
HDL-C (mmol/L)	1.11 ± 0.17	1.01 ± 0.25	NS
LDL-C (mmol/L)	2.16 ± 0.68	2.27 ± 0.90	NS
Creatinine (µmol/L)	63.02 ± 12.86	75.73 ± 15.91	NS
CRP (mg/L)	1.45 ± 1.01	2.45 ± 1.67	NS
Medications, n (%)			
Insulin or OAD	—	8(100)	
Statin	—	6(75)	
Aspirin	—	5(62.5)	

The data are given as the mean ± SD. or number of patients. CHD: coronary heart disease; BMI: body mass index; SBP: systolic blood pressure; DBP: diastolic blood pressure; GLU: fasting glucose; TC: total cholesterol; TG: total triglycerides; LDL-C: low-density lipoprotein cholesterol; HDL-C: high-density lipoprotein cholesterol; CRP: C-reactive protein; OAD: oral antidiabetic drugs.

Written informed consent was obtained from each patient. This study was approved by the Ethics Committee of the Liyuan Hospital and conformed to the Declaration of Helsinki (2008).

### Animals and treatments

Male C57BL/6 mice and ApoE**−/−** mice with a C57BL/6 background were purchased from Jackson Laboratory (Bar Harbor, ME, USA). These mice were bred and maintained in the Animal Center of Beijing University. These mice were maintained in a specific pathogen-free facility (Animal Center of Tongji Medical College of Huazhong University of Science and Technology, Wuhan, China). The 8-week-old ApoE**−/−** mice were fed a Western-type diet containing 21% fat and 0.15% cholesterol. All animal studies conformed to the principles of the National Institutes of Health Guide for the Care and Use of Laboratory Animals (NIH publication no. 85–23, revised 1996). All the protocols in this study were approved by the Animal Care and Use Committee of the Union Hospital of Huazhong University of Science and Technology, China. ApoE**−/−** mice were divided into two groups: one group was injected i.p. once per week for 10 weeks with 1 μg of recombinant human IL-37; the other group served as the control and was treated with PBS in the same manner. Euthanasia was performed by CO2 or isoflurane inhalation followed by heart removal.

To investigate whether the Treg response mediated the atheroprotective effect of IL-37, the ApoE**−/−** mice treated with or without IL-37 were randomly grouped and injected i.p. once per week for 10 weeks with anti-IL-10R mAb (200 μg) or isotype antibody.

### Weight and lipids measurement

The weight of each mouse was recorded 0 and 10 weeks following the start of the Western-type diet. The plasma was isolated from the blood of mice by centrifugation at 1200 *g* for 10 minutes after clotting at room temperature. The total cholesterol, triglyceride and high-density lipoprotein cholesterol levels were measured using enzymatic assays and determined using an autoanalyzer (Hitachi 917).

### Atherosclerotic lesion measurement

The atherosclerotic lesions were quantified in en face preparations of the whole aorta, and the frozen histological sections of the aortic sinus were processed as previously described^25, 26^. After the en face aorta lesion staining, the whole vessel images were captured using a digital camera. After the aortic sinus oil-red staining, all the images were collected and analyzed using the Image-Pro Plus 6.0 software.

For the lesions’ immunohistochemical analysis, approximately 5 μm sections of the aortic sinus were prepared. The antibodies used were as follows: purified anti-α-SMA antibody (1:200) for VSMCs, purified anti-monocyte/macrophage-2 (MOMA-2) (1:200) for monocytes and macrophages, and purified anti-CD4 antibody (1:50) for T cells. Masson’s trichrome was used for the detection of collagen in plaque area. The macrophages, VSMCs and collagen were quantified by assessing the percent positive area of total plague for each marker, and the CD4^+^ T cells were assessed by counting the number of cells stained positive per meter squared in the plaque area.

### Bone marrow-derived DC (BM-DC) generation

Bone marrow–derived DCs were generated as previously described^15, 16^. In brief, bone marrow was isolated from the C57BL/6 mice. The cells were depleted of red blood cells and were cultured with RPMI 1640 for 6 days in tissue culture plates at 37 °C and 5.0% CO_2_; the 1640 culture medium was supplemented with 10% FCS, 100 U/ml of penicillin, 100 U/ml of streptomycin, 20 ng/ml of granulocyte-macrophage colony-stimulating factor, and 10 ng/ml of IL-4. The purification of the DCs from the differentiated bone marrow cells was performed using a CD11c magnetic cell-sorting kit (Miltenyi Biotec). After purification, the DCs were exposed (48 h) to 20 µg/ml of oxidized low-density lipoprotein (oxLDL) or oxLDL plus 30 ng/ml of IL-37 in 1640. Cultured supernatant was collected for cytokine analysis. The cultured DCs were subsequently subjected to flow cytometry or co-culture.

### Cell isolation and preparation

The fresh spleens were removed from the mice and were gently squeezed with sterile needles in the cold PBS and passed through a stainless steel mesh screen; thus, the single-cell suspension was prepared. The CD4^+^ T cells were purified from splenocytes of C57BL/6 mice through a CD4^+^ T-cell isolation Kit (Miltenyi Biotec) and suspended at a density of 2 × 10^6^ cells/ml in complete culture medium RPMI 1640. The cells were subsequently subjected to flow cytometry or co-culture.

To observe the regulatory effects of IL-37 on the Th1/Th2/Th17/Treg paradigm *in vitro*, the CD4^+^ T cells (1 × 10^6^ cells/ml) were cocultured with oxLDL- treated BMDCs (2.5 × 10^5^ cells/ml), oxLDL plus IL-37-treated BMDCs (2.5 × 10^5^ cells/ml), or alone for 4 days in the presence of 2 μg/ml of anti-CD3 antibody. For cell staining, the cultured cells were then collected to measure the frequencies of Treg or were stimulated to measure the frequencies of Th1, Th2 and Th17 using a FACSCalibur flow cytometer. For the cytokine analysis, the cultured supernatant was collected.

### Flow cytometry

To analyze Th1 (CD4+IFN-γ+), Th2 (CD4+IL-4+), and Th17 (CD4+IL-17+), the splenic lymphocytes were stimulated with phorbol myristate acetate (PMA, 20 ng/ml), ionomycin (1 μg/ml) and monensin (2 μmol/ml). The incubator was set at 37 °C under a 5% CO_2_ environment. After 4 hours of culture, the cells were collected for staining according to the manufacturer’s instructions. Fixation and permeabilization were necessary prior to staining with IFN-γ, IL-4, or IL-17 antibody. To analyze the T regulatory cells, the cells were stained with anti-CD4-FITC and anti-CD25-APC Abs, followed by staining with anti-Foxp3-PE Ab after fixation and permeabilization according to the manufacturer’s instructions. Isotype controls were used to correct for compensation and confirm the antibody specificity.

To analyze the phenotype of splenic DCs, spleen was dissociated into single-cell suspensions. The cells were stained with CD11c, CD86 and MHC-II for 30 min.

Flow cytometry was performed using a FACSCalibur (BD Immunocytometry Systems, USA) and all analyses, including MFI (mean fluorescence intensity), were performed using FlowJo software (Treestar Inc., OR, USA).

### Real time polymerase chain reaction analysis

The aorta was extracted using TRIzol reagent (Invitrogen, USA) according to the manufacturer’s instructions. The total RNA was reverse transcribed using the RNA PCR kit (Takara Biotechnology, Dalian, China). The mRNA was analyzed via real-time PCR using the ABI PRISM 7900 Sequence Detector system (Applied Biosystems, USA) according to the manufacturer’s instructions. All the reactions were performed in duplicate for each sample. The relative mRNA expression level was calculated using the comparative CT method formula 2^−ΔΔCT^. The data were normalized to GAPDH. The primer pairs can be found as Supplementary Table S1.

### Cytokine and antibody assays

The plasma TGF-β1 levels were measured using an enzyme-linked immunosorbent assay (ELISA) according to the manufacturer’s instructions (eBioscience). The plasma levels of the other cytokines were measured using a Quantibody Mouse Interleukin Array 1 from Ray Biotech (Norcross, GA).

The cultured supernatant was measured using IFN-γ, IL-1β, IL-4, IL-6, IL-10, IL-12p70, IL-17 and TGF-β1 ELISA kits according to the manufacturer’s instructions.

To quantify anti-IL-10R-IgG or IL-37 specific antibodies, plates were coated with 100 µg/mL anti-IL-10R-IgG or IL-37, washed, and blocked. And then plasma harvested from mice was added at an optimized 1:50 dilution, and specific detection antibodies for mouse IgG were added (ELISA kit, eBioscience). The OD value was read at 450 nm.

### Statistical analysis

The data are shown as the means ± SEM. Comparisons between 2 groups were performed using Student’s *t* test when the data were normally distributed and the group variances were equal. The Mann–Whitney rank sum test was used when the data were not normally distributed or if the group variances were unequal. A one-way ANOVA was used for multiple comparisons among 3 groups, followed by the Bonferroni test when the data were normally distributed and group variances were equal. The Kruskal-Wallis test followed by the Dunn test was used when the group data were not normally distributed or if the group variances were unequal. All the statistical analyses were performed using the GraphPad Prism 6.0 software. P < 0.05 was considered to indicate significance.

## Electronic supplementary material

Supplementary Informations
