# Supplementary material for: Exogenous interleukin 37 ameliorates atherosclerosis via inducing the Treg response in ApoE-deficient mice
Source: Sci Rep. 2017 Jun 12;7:3310. doi: 10.1038/s41598-017-02987-4 (PMC5468328; doi:10.1038/s41598-017-02987-4)

**Exogenous** **interleukin 37** [**ameliorates atherosclerosis**](http://jem.rupress.org/content/205/2/339.abstract) **via** **inducing** **the Treg response in ApoE-deficient mice**

**Qingwei Ji^a,1^,** **Kai Meng^b,1^,** **Kunwu Yu^b,1^,** **Song Huang^c^** **Ying Huang^d^,** **Xiaohong Min^e^,** **Yucheng Zhong^b^,** **Bangwei Wu^b^,** **Yuzhou Liu^b^,** **Shaoping Nie^f^, Jianwei Zhang^a^,** **Yujie Zhou^a,★^, Qiutang Zeng^b,★^**

^a^**Department of Cardiology, Beijing Anzhen Hospital, Capital Medical University, Beijing Institute of Heart Lung and Blood Vessel Disease, The Key Laboratory of Remodeling-related Cardiovascular Disease, Ministry of Education, Beijing 100029, China;** **^b^Laboratory of Cardiovascular Immunology, Institute of Cardiology, Union Hospital, Tongji Medical College, Huazhong University of Science and Technology, Wuhan, China;** **^c^****Department of Orthopedics, Liyuan Hospital, Tongji Medical College, Huazhong University of Science and Technology, Wuhan, China;** ^d^**Department of Ultrasound, the People’s Hospital of Guangxi Zhuang Autonomous Region, Nanning 530021, China;** **^e^Department of Pathology, Puren Hospital, Wuhan University of Science and Technology, Wuhan, China; ^f^Emergency & Critical Care Center, Beijing Anzhen Hospital, Capital Medical University, Beijing Institute of Heart Lung and Blood Vessel Disease, The Key Laboratory of Remodeling-related Cardiovascular Disease, Ministry of Education, Beijing 100029, China.**

**^1^These authors contributed equally to this work**

**This author takes responsibility for all aspects of the reliability and freedom**

**from bias of the data presented and their discussed interpretation**

**^★^Corresponding author: Dr. Qiutang Zeng, Laboratory of Cardiovascular Immunology,** **Institute of Cardiology, Union Hospital, Tongji Medical College, Huazhong University of Science and Technology, Wuhan, China**

**Tel:** **86-27-85726432 Fax: 86-27-85726432 Email:** [**zengqiutang007@163.com**](mailto:zengqiutang007@163.com)

**^★^Corresponding author: Dr. Yujie Zhou, Department of Cardiology, Beijing Anzhen Hospital, Capital Medical University, Beijing Institute of Heart Lung and Blood Vessel Disease, The Key Laboratory of Remodeling-related Cardiovascular Disease, Ministry of Education, Beijing 100029, China**

**Tel: 86-10-64456489 Fax: 86-10-64442234 Email:** [**azzyj12@163.com**](mailto:azzyj12@163.com)

**Figure S1.** Detecting IL-37 expression in human atherosclerotic plaques using immunofluorescence. A, The co-expression of IL-37 and macrophages identified by anti-CD68 antibody. B, The co-expression of IL-37 and VSMCs identified by anti-α-SMA. C, The co-expression of IL-37 expression and T lymphocytes identified by anti-CD3 antibody.

**Figure S2.** Ox-LDL almost did not induce apoptosis of dendritic cells (DCs). The apoptosis of DCs was detected by FACS using Annexin-v and PI. Isotype, PBS, or Ox-LDL with 20 µg/ml was added in DCs respectively for 48h.

**Figure S3.** IL-37 inhibited the maturation of BMDCs by LPS in vitro. Mean fluorescence intensity (MFI) of CD86 and MHC-II. BMDCs were treated with PBS, LPS(1 µg/ml), or IL-37 (30 ng/ml)+ LPS(1 µg/ml) respectively for 48h.

**Figure S4.** Levels of plasma neutralizing antibodies of human IL-37 for PBS and IL-37 group. Values are presented as the mean±SEM, n=6. # p>0.05.

**Figure S5.** Levels of plasma neutralizing antibodies of human IL-37 for different groups, including PBS+lgG, IL-37+lgG, PBS+anti-IL10R, IL-37+anti-IL10R. n=6. # p>0.05.

**Table S1**

Table S1 Real-Time PCR Primer Sequences

| Molecule | Sequence (5’-3’) |
| --- | --- |
| IFN-γ forward | ACTGGCAAAAGGATGGTGAC |
| IFN-γ reverse | TGAGCTCATTGAATGCTTGG |
| TBX21 forward | ATTGGTTGGAGAGGAAGCGG |
| TBX21 reverse | TGTGCACCCTTCAAACCCTT |
| IL-12 forward | ATCGTTTTGCTGGTGTCTCC |
| IL-12 reverse | CTTTGTGGCAGGTGTACTGG |
| IL-4 forward | ACGAGGTCACAGGAGAAGGGA |
| IL-4 reverse | AGCCCTACAGACGAGCTCACTC |
| GATA3 forward | GCTACGGTGCAGAGGTATCC |
| GATA3 reverse | TTCACACACTCCCTGCCTTC |
| IL-1β forward | GGGCCTCAAAGGAAAGAATC |
| IL-1β reverse | TACCAGTTGGGGAACTCTGC |
| IL-6 forward | AGTTGCCTTCTTGGGACTGA |
| IL-6 reverse | TCCACGATTTCCCAGAGAAC |
| IL-17 forward | TCCAGAAGGCCCTCAGACTA |
| IL-17 reverse | AGCATCTTCTCGACCCTGAA |
| RORγT forward | CTGTCCTGGGCTACCCTACT |
| RORγT reverse | CCACTTGTTCCTGTTGCTGC |
| IL-23 forward | AATAATGTGCCCCGTATCCA |
| IL-23 reverse | CATGGGGCTATCAGGGAGTA |
| TGF-β1 forward | TGCTTCAGCTCCACAGAGAA |
| TGF-β1 reverse | TGGTTGTAGAGGGCAAGGAC |
| IL-10 forward | ATAACTGCACCCACTTCCCA |
| IL-10 reverse | GGGCATCACTTCTACCAGGT |
| FOXP3 forward | CACCTATGCCACCCTTATCC |
| FOXP3 reverse | CGAACATGCGAGTAAACCAA |
| GAPDH forward | AACTTTGGCATTGTGGAAGG |
| GAPDH reverse | CACATTGGGGGTAGGAACAC |

All these primers were synthesized by Invitrogen in Shanghai. IFN indicates interferon; IL, interleukin; TGF, transforming growth factor; MMP, indicates matrix metallo preteinases.

**Figure S1**





**Figure S2**


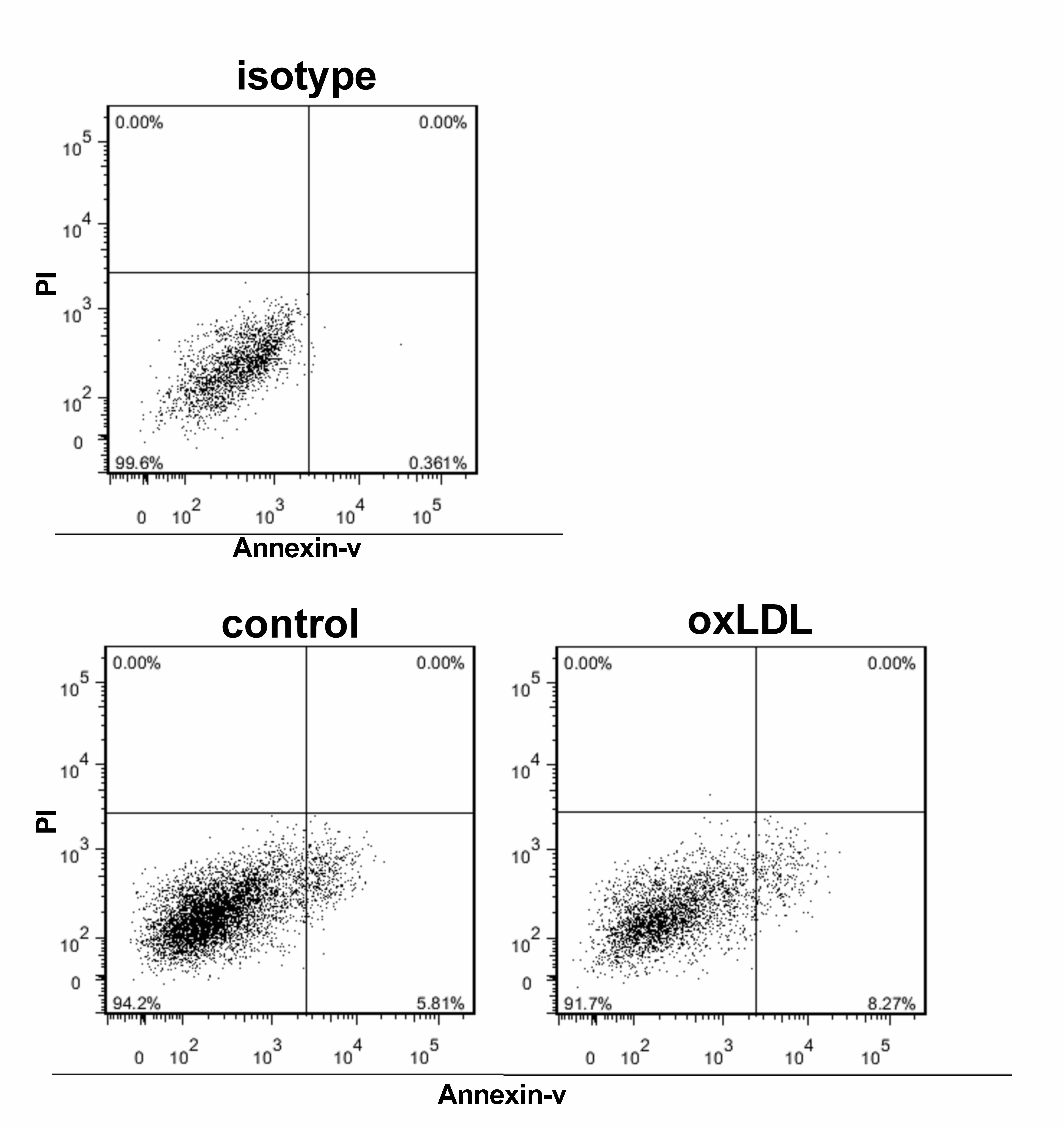


**Figure S3**


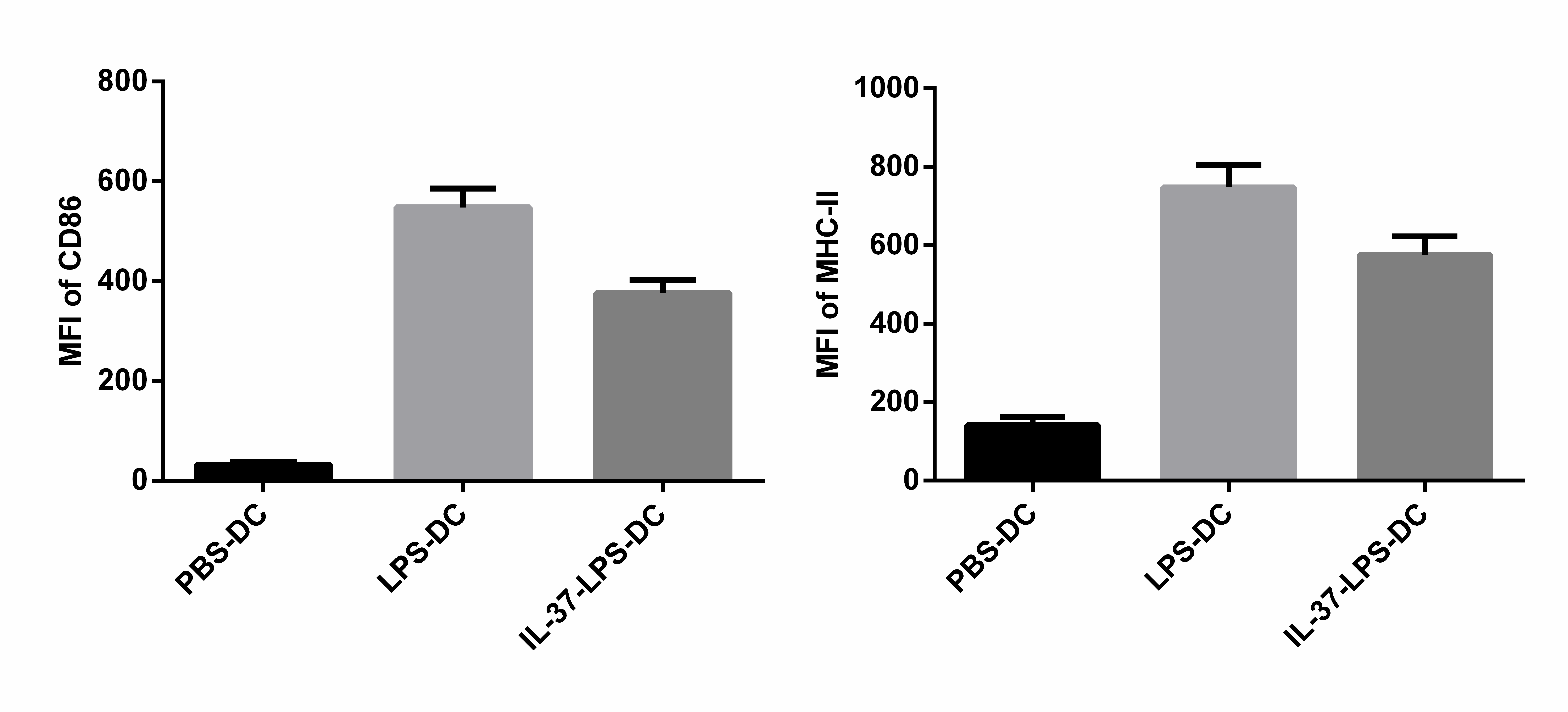


**Figure S4**


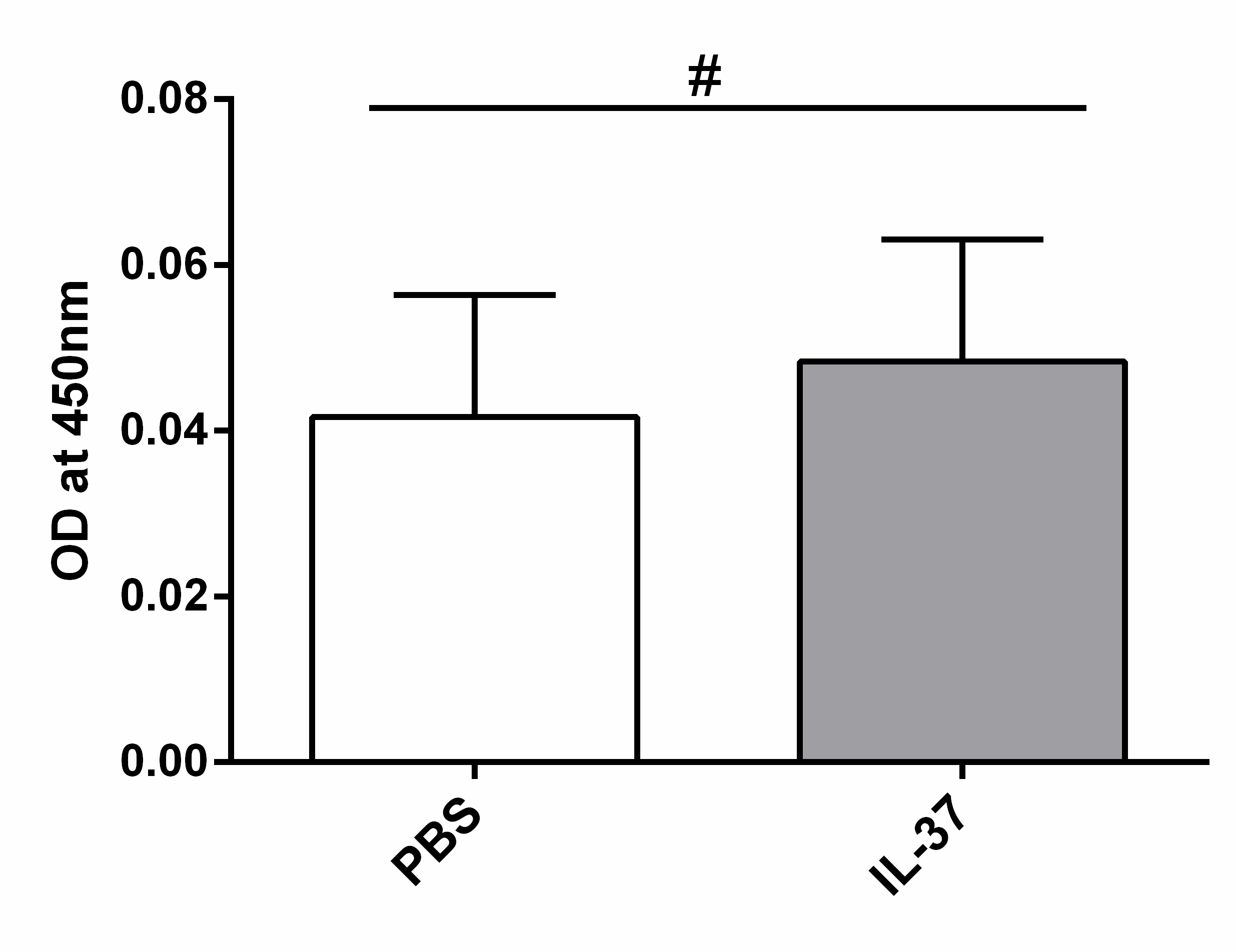


**Figure S5**


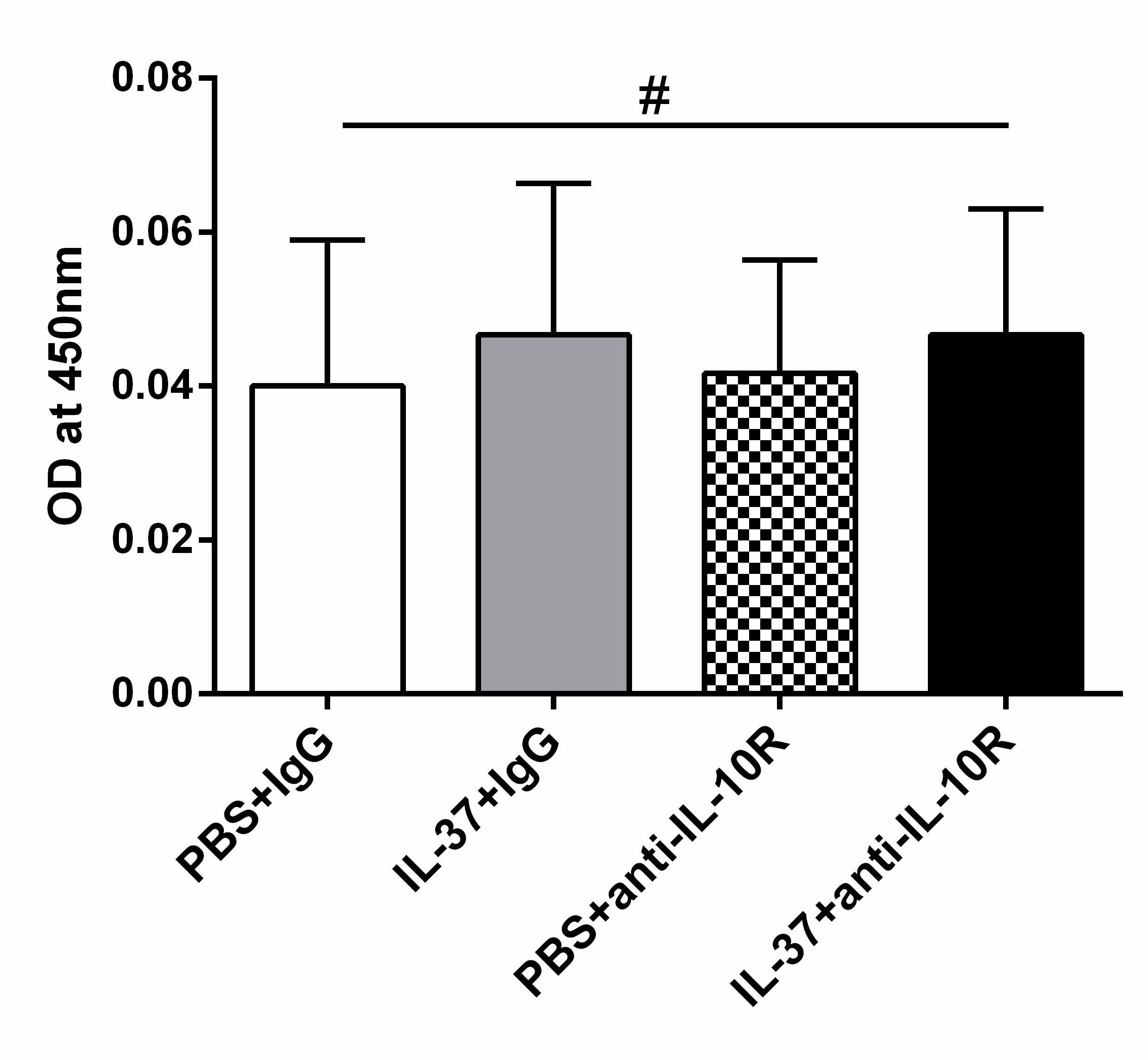

Supplement: Supplementary file 1 — Supplementary Informations [file 41598_2017_2987_MOESM1_ESM.doc]
